# A Fiber-Coupled Stimulated Emission Depletion Microscope for Bend-Insensitive Through-Fiber Imaging

**DOI:** 10.1038/s41598-019-47319-w

**Published:** 2019-07-31

**Authors:** Brendan M. Heffernan, Stephanie A. Meyer, Diego Restrepo, Mark E. Siemens, Emily A. Gibson, Juliet T. Gopinath

**Affiliations:** 1 Department of Physics, University of Colorado Boulder, Boulder, CO 80309 USA; 2 Department of Bioengineering, University of Colorado Anschutz Medical Campus, Aurora, CO 80045 USA; 3 Department of Cell and Developmental Biology, University of Colorado Anschutz Medical Campus, Aurora, CO 80045 USA; 4 Department of Physics and Astronomy, University of Denver, Denver, CO 80210 USA; 5 Department of Electrical, Computer, and Energy Engineering, University of Colorado Boulder, Boulder, CO 80309 USA

**Keywords:** Super-resolution microscopy, Fluorescence imaging, Imaging techniques

## Abstract

We present results for a new type of fiber-coupled stimulated emission depletion (STED) microscope which uses a single fiber to transport STED and excitation light, as well as collect the fluorescence signal. Our method utilizes two higher-order eigenmodes of polarization maintaining (PM) fiber to generate the doughnut-shaped STED beam. The modes are excited with separate beams that share no temporal coherence, yielding output that is independent of fiber bending. We measured the resolution using 45 nm fluorescent beads and found a median bead image size of 116 nm. This resolution does not change as function of fiber bending radius, demonstrating robust operation. We report, for the first time, STED images of fixed biological samples collected in the epi-direction through fiber. Our microscope design shows promise for future use in super-resolution micro-endoscopes and *in vivo* neural imaging in awake and freely-behaving animals.

## Introduction

Stimulated emission depletion (STED) microscopy has become an indispensable tool for studying biological systems because it achieves resolutions an order of magnitude better than conventional diffraction-limited confocal microscopy^1,2^. In STED microscopy, a fluorescence excitation laser beam is overlapped with a spatially-structured STED laser beam. The wavelength of the STED laser is tuned to the tail of the fluorescence emission spectrum of the sample to deplete the fluorescence and hence create a smaller effective area of illumination. For improved lateral resolution, the STED beam passes through a phase plate or spatial light modulator (SLM) to produce a vortex phase that results in a doughnut shaped intensity profile with a characteristic central null. The resolution is improved because the fluorescence signal is suppressed everywhere except the dark center. STED microscopy requires no post-acquisition computational processing so the rate at which images can be acquired is limited only by the scanning speed, number of pixels, and the desired signal to noise ratio^3,4^. This has resulted in video rate super-resolution microscopy, which is perfect for studying live-cell dynamics in real time with unprecedented detail^5–9^.

An exciting area of research facilitated by video-rate STED is the study of the dendritic spines of neurons, which change their morphology over time in response to the environment. These changes are thought to play an important role in learning and memory, but occur on length scales that are sub-diffraction limited, necessitating the usage of super-resolution microscopy^10^. Due to their environmental dependence, dendritic spines are best studied *in vivo*^3^. To that end, STED and two-photon STED (2P-STED) have been used to image spine formation in alive, but anesthetized and restrained mice over extended time periods^11–13^. Ideally, the animal would be awake and free to move, so that changes in morphology associated with learning and behavior could be visualized in real time. Recently, researchers have demonstrated miniature, head-mounted, fiber-coupled two-photon microscopes (without STED) to image the brain of an awake and freely-moving mouse, but the resolution of this method is diffraction-limited and cannot resolve many important features^14–17^. Observing neuronal activity in an awake animal is crucial to further understanding the brain; combining STED and a miniature head-mounted microscope would provide unprecedented resolution and therefore groundbreaking new opportunities for neuroscience studies. Ideally, this miniature, fiber-coupled microscope would transport STED and excitation light to the sample through a single fiber and collect fluorescence in the epi-direction through the same fiber (through-fiber imaging).

This goal presents many challenges including: designing a microendoscopic objective with high NA and minimal aberration, correcting for movement artifacts, and achieving reasonable penetration depth through brain tissue. Here, we propose a solution to the problem of transporting excitation, fluorescence and a shaped STED beam through a single fiber in a robust manner. The primary obstacle to fiber-based STED is that standard, commercially available step index fiber supports doughnut-shaped modes that are very nearly degenerate, and random perturbations can transfer power from one mode to another^18^. These modes then interfere to produce an output that is generally not a doughnut. This means that while it is possible to achieve a doughnut beam after propagating through the fiber, the doughnut is highly condition-dependent and any change, i.e. bending or heating of the fiber, will result in a modified output spatial mode. Obviously, this is unacceptable for applications in live animal imaging.

**Figure 1:**
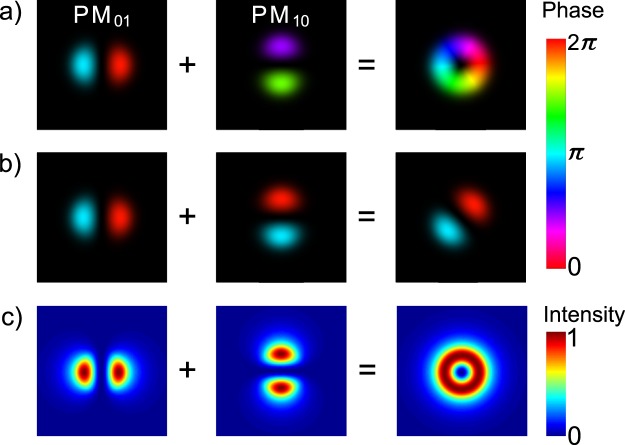
Simulated addition of PM fiber modes. (**a**) and (**b**) When the two excited fiber modes are coherent with respect to each other, i.e. their phases are correlated, the resulting mode depends on their relative phase. This means that fiber bending will change the output by changing the relative phase. (**c**) If the two modes share no phase correlation, the resulting mode is a doughnut shape regardless of relative phase^19–21^.

An attractive solution is offered by doughnut modes with orbital angular momentum (OAM), generated by selectively exciting higher order modes of a polarization maintaining (PM) fiber^19–21^. This method requires coupling light to two orthogonally oriented eigenmodes which are well-described by Hermite-Gaussians (HG) and depicted in Fig. 1. We refer to them as *PM*_01_ and *PM*_10_. If they are excited by two laser beams that are mutually coherent, the output will be sensitive to the relative phase between them (Fig. 1a,b), and so the output will be sensitive to fiber bending and heating^19,20^. Fortunately, the output sensitivity can be mitigated by removing the temporal coherence between the two modes. *If two PM fiber modes are excited with lasers that are mutually incoherent, the output of the fiber remains a doughnut intensity pattern regardless of fiber bending, because the two modes do not interfere* (Fig. 1c)^21^. This is the key principle that gives our fiber STED microscope robust operation across varying conditions. Note that the resulting doughnut beam does not contain OAM, as the phases of the two added modes have no correlation and therefore, no helical structure. This type of STED beam, essentially the overlap of two Hermite-Gaussian modes, was reported in early STED literature, but this is the first time it has been applied to build a fiber STED microscope^22^.

Alternative approaches to fiber STED have been proposed and demonstrated previously. One solution is to use customized “vortex” fiber that supports doughnut eigenmodes. An all-fiber STED system based on this solution maintains high-purity modes suitable for STED even under extreme bending, with resolutions of 103 nm^23,24^. Another solution uses double-clad fiber and two-photon excitation, and has demonstrated through-fiber imaging of fluorescent beads, but these experiments were performed at a relatively low numerical aperture (NA) of 0.35, resulting in a STED resolution of 310 nm^25^. Although in principle this approach could scale to higher NA, it has not been demonstrated. It is also not clear that this implementation is fiber-condition independent because no characterization of robustness with respect to fiber bending was made. Neither of these methods have been able to achieve through-fiber imaging of biological samples, a key benchmark for transitioning fiber STED technology into endoscopic applications. Therefore, our approach represents a key breakthrough in fiber STED.

In this paper, we demonstrate a proof-of-concept fiber STED microscope that utilizes the *PM*_01_ and *PM*_10_ modes of a commercially available PM fiber to generate a doughnut STED beam. By exciting these two fiber modes using two beams that are temporally incoherent with respect to each other, we establish robust operation with no loss of resolution due to fiber bending. We acquire through-fiber STED images of fluorescent beads and, for the first time, biological samples. These images indicate a typical resolution of 116 nm.

## Results

### Fiber STED microscope design

**Figure 2:**
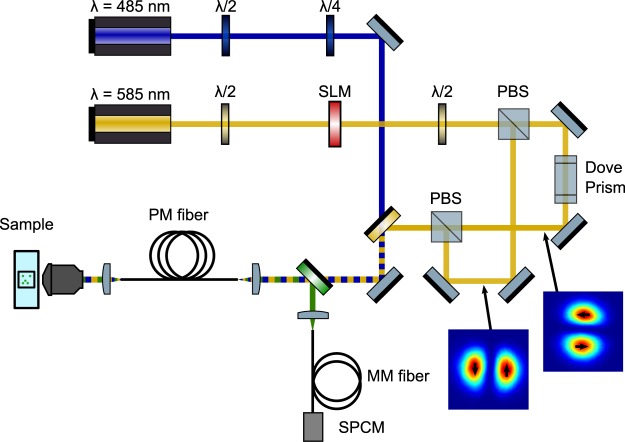
A diagram of the fiber STED microscope. Fluorophores are excited at 485 nm and depleted at 585 nm. The lasers have a repetition rate of 20 MHz. The STED laser (at 585 nm) is shaped to match the *PM*_01_ mode using a spatial light modulator (SLM). A polarizing cube beamsplitter (PBS) splits the beam into a Mach-Zehnder interferometer. In one arm, a dove prism rotates the spatial profile of the laser and introduces a delay of 74 ps. The transverse modes of the two STED beams are pictured, with the polarization indicated by black arrows. These beams are combined using another PBS and coupled to PM fiber (two meter length). The excitation light is sent through quarter and half-wave plates to control its polarization and then a high-pass dichroic mirror combines it with the STED light. Upon exiting the fiber, the STED and excitation beams are collimated and focused onto a fluorescent sample using a 1.4 NA, oil-immersion objective. This objective collects and collimates fluorescence, which is then coupled back into the PM fiber. A dichroic mirror splits the fluorescence from the common beam path. The light is passed through two chromatic filters and then focused into a multimode (MM) fiber coupled to a single photon counting module (SPCM).

Our custom microscope was constructed almost entirely from off-the-shelf parts and is similar in design to typical confocal fluorescence microscopes (Fig. 2). We excite fluorescence using 500 ps pulses at 485 nm (PicoQuant LDH-P-C-485B). Excitation pulses are temporally overlapped with 1 ns pulses of STED light at 585 nm (Mobius Photonics Rainbow-7–20MHz). Both lasers have a repetition rate of 20 MHz. To efficiently couple light into the fiber, the depletion laser is shaped to match the *PM*_01_ mode using a spatial light modulator (Meadowlark Optics, Standard 1920 × 1152 Nematic SLM System). The STED beam then passes through a half-wave plate, and a polarizing cube beam splitter (PBS) separates it into two arms of a Mach-Zehnder interferometer. In one arm, a Dove prism rotates the spatial profile by 90 degrees and delays the pulse by approximately 74 ps. The coherence time for the STED laser is estimated from the laser linewidth to be 2 ps. A second PBS recombines the two STED beams. A quarter-wave plate followed by a half-wave plate controls the polarization of the excitation beam, and a high-pass dichroic mirror (Chroma ZT561sprdc) combines it with the STED beam path. All three beams are coupled into a loosely coiled optical fiber (Thorlabs P1-980PM-FC-2) with a bend radius of approximately 50 mm. The fiber supports 6 modes at 585 nm; three different transverse profiles with two possible polarizations for each transverse mode. Twelve modes are supported at 485 nm. Care is taken to couple the excitation light into a Gaussian-like mode, which propagates without intermodal coupling over the 2 m fiber length. An apochromatic objective (Mitutoyo 10x plan apochromat) collimates the output of the fiber, which is then directed into an oil immersion, 100X, 1.4 NA microscope objective (Olympus UPLSAPO 100XO). A piezo stage (Mad City Labs Nano-LP100 XYZ) raster scans the sample in three dimensions through the focus.

The high NA objective collects and collimates fluorescence from the sample, which then retraces the path of the STED and excitation beams before being coupled into the PM fiber. The counterpropagating fluorescence is collimated after exiting the fiber and split from the beam path using a dichroic mirror (Chroma T525/50dcrb) that reflects fluorescent wavelengths. The light is then passed through two chromatic filters (Semrock SP01-561RU-25 and FF01-520/35-25) so as to detect only fluorescent light. A fiber-coupled single photon counting module (SPCM)(Excelitas SPCM-AQR-15) detects and counts fluorescent photons for a variable exposure/pixel dwell time, typically 50 microseconds.

### Characterizing system resolution

**Figure 3:**
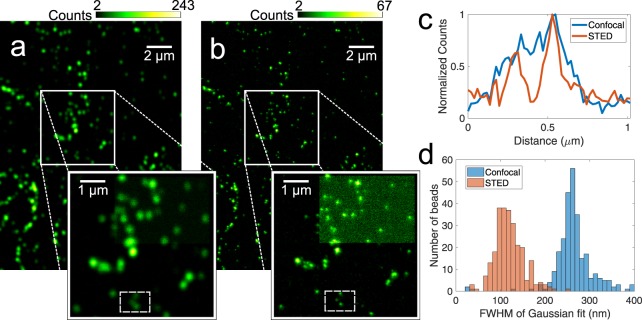
Images of 45 nm fluorescent beads attained using (**a**) confocal and (**b**) STED modality. There was approximately 45 mW of power in the STED laser before the objective. The pixels size is 19.5 nm. These images have been convolved with a small Gaussian (waist of 0.8 pixels) for smoothing and the background has been subtracted to enhance clarity. A magnified area of interest is shown, and (**c**) a cross sectional cut of the boxed region within the magnification is shown. The top right corner of the magnified area in (**a**) and (**b**) shows raw confocal and STED images from the microscope for comparison. (**d**) Histogram of Gaussian-fitted FWHM values for bead images. The confocal distribution has a median of 260 nm, while the STED distribution has a median of 116 nm. This demonstrates more than a two-fold improvement of resolution from confocal. Both (**c**) and (**d**) were derived from raw data.

Proof-of-concept images showing more than a two-fold improvement in resolution compared to confocal modality were attained from both fluorescent test targets and biological samples. Images of 45 ± 6 nm fluorescent beads (Invitrogen FluoSpheres F8795) were taken using approximately 45 mW of STED light and ≈7 *μ*W of excitation, as measured before the objective (Fig. 3). A clear improvement in resolution over confocal imaging can be seen, with closely spaced beads becoming distinguishable when using STED. The resolution of a STED system depends on the properties of the fluorphore in use and on the conditions to which the molecules are subjected^1,26^. This makes it difficult to assign a resolution to the STED system. However, as an estimate, we fit Gaussians to the images of the fluorescent beads and define the resolution as the full width half max (FWHM) of the fitted Gaussian. This is done algorithmically using a peak-finding code to define a region of interest centered on a bead. Peaks that are too close together are omitted in order to get accurate fits. This yields distributions of FWHM values, shown in Fig. 3(d). We take the median of this distribution as the typical resolution. This procedure gives an estimated resolution of 260 ± 7 nm confocal and 116 ± 6 nm STED. We do not take into account the finite size of the beads, so the resolution derived from the FWHM of imaged beads represents an upper limit estimate of the true resolution of the microscope. The uncertainty in median values of these distributions is due to variations in the input parameters of the peak finding code that can produce slightly different outcomes.

**Figure 4:**
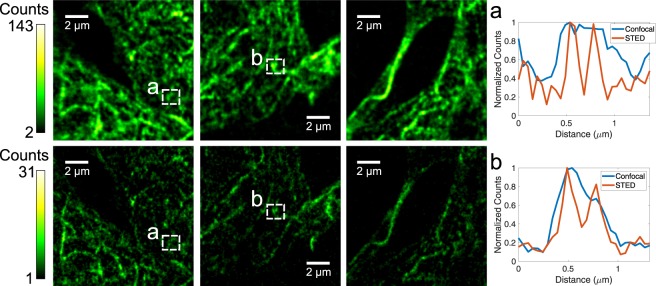
Images of HeLa cells immunostained for tubulin using Alexa 488. The images have been convolved with a Gaussian with a waist of 0.8 pixels (39 nm) for smoothing and the background was subtracted. The top row are confocal and the bottom row are STED images. Normalized linecuts of raw data are shown in (**a**) and (**b**), demonstrating at least a two-fold improvement in resolution. The pixel size is 48.8 nm and approximately 20 mW of STED and 6 *μ*W of excitation power were used, measured before the objective. Figure reproduced from^27^.

While images of fluorescent beads are well-suited for resolution estimation, the true test of a STED system is its ability to image biological samples. Thus, we collected images of tubulin immunostained with Alexa 488 in fixed HeLa cells through fiber (Fig. 4). Compared to confocal, a clear improvement in resolution is seen, with features and their morphologies becoming more distinguishable in the STED case, including structures as small as 120 nm. To the best of our knowledge, this represents the first successful through-fiber STED image of a biological sample.

We have also experimentally demonstrated that this method of fiber STED yields fiber-condition-independent resolutions. This robustness is essential for practical deployment of fiber STED for *in vivo* applications, such as the study of brain function in freely moving animals. Theoretically, the STED beam should be insensitive to fiber conditions in the limit that the two fiber modes making up the STED doughnut are completely incoherent with respect to one another. As an initial test, the two STED beams were coupled into the fundamental mode of the PM fiber and the interferometric visibility was measured. A piezo delay stage was used to scan the relative phase between the two beams, resulting in a fringe visibility of 3%. This indicates very minor mutual coherence between the two STED modes and that the output of the fiber will not change with bending or heating.

**Figure 5:**
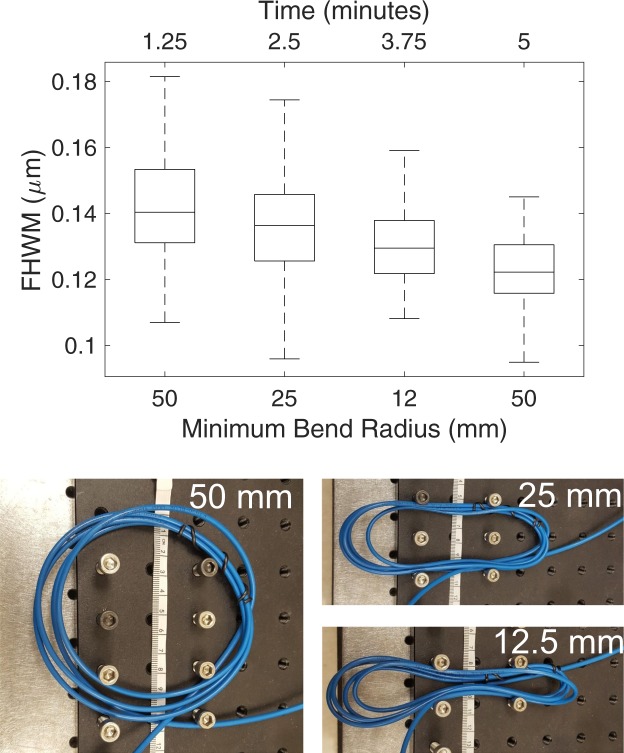
Box plot of the measured FWHM of bead images for 100 nm fluorescent beads. The center line in each box gives the median of the data set and the outer edges of the box represent the 25th and 75th percentile. The dashed line and bar denote the extremes of the distribution, not considering statistical outliers. Note that while the distribution of FWHM’s changes, this is caused by a time drift in the z-axis of the stage rather than by changes in fiber conditions. Below are images of the fiber in bent states, with minimum bend radius marked. Figure reproduced from^27^.

Next, we implemented a more rigorous test wherein a 13 × 20 *μ*m field of view containing 100 nm fluorescent beads (Invitrogen FluoSpheres F8803) was imaged four times in a row, as the fiber was bent into different configurations between frames. Each image took approximately 75 seconds to acquire due to scan speed limitations, and the total time elapsed for the measurement was 5 minutes. The first frame was taken with the fiber in its typical resting position (50 mm bend radius), as was the last to control for photobleaching, drift of the piezo stage in the z direction, or any drift in alignment into the fiber that could occur during the time lapse between acquisitions. For each frame, single beads were detected and fitted to a Gaussian as above, resulting in a distribution of FWHM values made up of at least 142 beads. The distribution was found to change over the course of the measurement due to one of these drift effects, but not bending (Fig. 5). This conclusion is inferred from the observation that the resting bend radius of the fiber does not produce the same results at the beginning and end of the measurement. The test demonstrates that our approach to fiber STED microscopy produces a robust platform, in agreement with theoretical considerations.

## Discussion

We have demonstrated a bend-independent fiber STED microscope capable of through-fiber imaging of fluorescent nanobeads and immunolabeled tubulin in HeLa cells. The key that enables such a system is the use of two, temporally-incoherent STED laser modes that couple to the *PM*_01_ and *PM*_10_ modes of a PM fiber to create a bend insensitive STED doughnut. The fiber STED microscope has an estimated resolution of 116 nm, which is currently limited by available STED laser power.

The polarization of the STED modes must be taken into account in order to ensure that the doughnut beam maintains its dark center upon high NA focusing. It is well known that tight focusing of doughtnut beams possessing OAM can cause photonic spin-orbit interaction and collapse the central null of the beam^28–31^. Similarly, intensity can leak into the zero-intensity lines of the PM fiber modes if the polarization is not chosen correctly. This can be shown by approximating the PM mode as a sum of two Laguerre-Gaussian (LG) modes with opposite values of l. The dark null can be preserved for linear polarization, but for circular polarization, one of these terms will be spin-orbit anti-aligned and so result in partial collapse. This process is a critical concern considering that the dark center is crucial for attaining sharp image contrast by preserving as much fluorescence signal as possible from the emitter^2,32,33^. We performed calculations of tight-focusing using the Debye-Wolf integral to verify that higher-order PM fiber modes should have polarization parallel to the line of zero intensity to focus properly, in agreement with the literature^22,26,34^. Polarization also plays a role for excitation light, which should be circularly polarized in order to optimally excite the fluorophores. We use quarter and half-waveplates to ensure the excitation light is circularly polarized after exiting the fiber. No change in output polarization is observed during bending.

Our strategy of using linear polarization for the STED doughnut has consequences for the operation of our microscope, as fluorophores can exhibit sensitivity to both excitation and STED polarization^22,26,35–38^. Consider a fluorophore with a given orientation of its dipole moment. It is optimally excited when the polarization of the excitation light is parallel to the dipole moment, with decreasing probability of excitation as a relative angle forms between them. The excitation light should therefore be circularly polarized in order to ensure that at some point during every cycle of light, the polarization and dipole moment are parallel. This argument can similarly be applied to the STED light causing de-excitation via stimulated emission. However, the fiber (STED) modes must remain linearly polarized to maintain a dark center, and so cannot deplete fluorophores as efficiently as circularly polarized STED beams across its full spatial extent. This can result in asymmetric images for single molecules, because fluorescence is not depleted in places where the STED beam is polarized perpendicular to the dipole moment of the molecule. This phenomenon has been leveraged in other STED microscopes to gain information about the orientation of molecules in a target, resulting in molecular orientation microscopy using STED (MOM-STED)^35^. This capability is fundamental to our microscope. However, we expect that the STED polarization constraint should not play a large role in situations where fluorophores are able to freely rotate, where there are many randomly oriented fluorophores, or for an objective with a smaller numerical aperture. In low NA situations, the polarization of the doughnut mode is far less crucial because the focus is not tight enough to collapse nodal features, and so in principle the STED polarization could be made circular.

Our STED microscope is still under development and many exciting avenues exist for the future. Foremost among these is improving the signal-to-noise ratio. There is a loss of signal in any fiber-coupled microscope due to reflections at the fiber facet and possible modal mismatch between incoming fluorescent light and the supported modes of the fiber. The issue of modal mismatch is especially important for few-mode fibers like those used in this work. Recently, double-clad fibers have been deployed in conventional fluorescence microscopes that allow for more efficient signal collection^39^. Equally important is reducing noise sources associated with fiber. Large backgrounds have been observed, possibly caused by laser pulses reflected from the fiber facet and collimated using the coupling lens. Another possible background source is autofluorescence within the fiber^40^. In the future, these noise sources might be mitigated through time correlation photon counting techniques or refined detection schemes. Resolution can be improved by increasing power in the STED beam, which is currently limited to about 45 mW, time averaged. Our fiber STED microscope can readily be made into an all-fiber implementation where the higher-order fiber modes are excited from the fundamental using long-period Bragg gratings to provide more robust and alignment-tolerant operation^41^. Our microscope also provides a basis for the creation of a fiber two-photon STED imaging system that would provide deep tissue super-resolution. This simple and robust fiber platform solves the problem of propagating the STED doughnut beam through fiber, enabling the future possibility of super-resolution, awake-behaving animal imaging when combined with a properly designed microendoscopic objective.

## Conclusions

We have demonstrated STED images of both fluorescent beads and biological samples through commercially available fiber. To the best of our knowledge, this is the first report of successful through-fiber STED imaging of biological samples. Median resolutions of 116 nm are achieved. We have shown that our approach yields bending-independent resolution, providing a robust platform for STED imaging. This is achieved by using two mutually incoherent beams coupled to higher-order modes of a polarization maintaining fiber. It is possible to extend this framework to fiber two-photon STED and even to an all-fiber implementation for coupling and detection. We believe our fiber STED microscope will greatly enhance the applicability of STED imaging and could provide a platform for flexible, *in vivo*, imaging of awake-behaving animals in the future.

## Methods

### Bead image analysis

Fluorescent beads with a diameter of 45 nm (Invitrogen FluoSpheres F8795) were imaged with a 20-by-20 *μ*m field of view in both confocal and STED modalities. The pixel dwell time was 50 *μ*s and pixel size was 19.5 nm. The left-hand-side of the images were warped due to a detector synchronization issue, and so cropped out for analysis. A peak-finding code written in Matlab was used to find regions of interest (ROI’s) centered on every bead in the image, which were then subjected to a least squares Gaussian curve fitting function along both x and y. Beads were not analyzed if they were too close together or to the edge of the image, as this yields inaccurate fits. Images were also inspected manually to confirm the accuracy of ROI’s. FWHM values were extracted from the fitted Gaussians and the x and y values were averaged. The result of this routine is shown in Fig. 3.

### Fluorescent bead sample preparation

The fluorescent bead solution was diluted with deionized water and sonicated for 10 minutes while polylysine was applied to a microscope coverslip and left for 2 minutes. The coverslip was rinsed with deionized water and the bead solution was pipetted on to the coverslip and left to adhere for 5 minutes. Afterward, the coverslip was rinsed again and mounted to a slide using ProLong (ThermoFisher Scientific P36930).

### HeLa cell sample preparation

The HeLa cells were fixed in 2 mL of 4% paraformaldehyde for 15 minutes at room temperature before being quenched for 5 minutes in 375 mg of glycine mixed with 50 mL 1X PBS. The cells were then permeabilized for 30 minutes at room temperature in 2 mL of incubation buffer (0.1 mg BSA, 200 mg saponin, 1 mL fetal bovine serum, and 50 mL 1x PBS). They were left to incubate in a humidified chamber for 1 hour at room temperature in 100 *μ*L of incubation buffer and purified anti-Tubulin *β*3 antibody. After being washed twice for five minutes each using 2 mL of incubation buffer, they were again incubated for thirty minutes in 100 *μ*L of incubation buffer and Alexa Fluor 488 AffiniPure donkey anti-mouse IgG. The washing step was repeated and the cells were mounted on a glass slide in Prolong Diamond.

## Data Availability

The datasets generated during and/or analysed during the current study are available from the corresponding author on reasonable request.
